# Relationship between betel quid chewing and radiographic alveolar bone loss among Taiwanese aboriginals: a retrospective study

**DOI:** 10.1186/1472-6831-14-133

**Published:** 2014-11-04

**Authors:** Chun-Nan Hsiao, Chun-Chan Ting, Tien-Yu Shieh, Edward Chengchuan Ko

**Affiliations:** School of Dentistry, College of Dental Medicine, Kaohsiung Medical University, Kaohsiung, Taiwan; Lab of Tissue Engineering, College of Dental Medicine, Kaohsiung Medical University, Kaohsiung, Taiwan; Department of Periodontology, College of Dentistry, Aichi-Gakuin University, Nagoya, Japan; Department of Oral Hygiene, College of Dental Medicine, Kaohsiung Medical University, Kaohsiung, Taiwan; Division of Oral and Maxillofacial Surgery, Kaohsiung Medical University Hospital, Kaohsiung, Taiwan; Department of Cartilage and Bone Regeneration (Fujisoft), Graduate School of Medicine, The University of Tokyo, 7-3-1 Hongo, Bunkyo, Tokyo, 113-8654 Japan

**Keywords:** Areca, Betel, Alveolar bone loss, Periodontitis

## Abstract

**Background:**

Betel quid chewing is associated with the periodontal status; however, results of epidemiological studies are inconsistent. To the best of our knowledge, no study has reported radiographic alveolar bone loss (RABL) associated with betel quid chewing.

**Methods:**

This survey was conducted in an aboriginal community in Taiwan because almost all betel quid chewers were city-dwelling cigarette smokers. In total, 114 subjects, aged 30–60 years, were included. Full-mouth intraoral RABL was retrospectively measured and adjusted for age, gender, and plaque index (PI). Multiple regression analysis was used to assess the relationship between RABL and potential risk factors.

**Results:**

Age-, gender-, and PI-adjusted mean RABL was significantly higher in chewers with or without cigarette smoking than in controls. Multiple regression analysis showed that the RABL for consumption of 100,000 pieces betel quid for the chewer group was 0.40 mm. Full-mouth plotted curves for adjusted mean RABL in the maxilla were similar between the chewer and control groups, suggesting that chemical effects were not the main factors affecting the association between betel quid chewing and the periodontal status.

**Conclusion:**

Betel quid chewing significantly increases RABL. The main contributory factors are age and oral hygiene; however, the major mechanism underlying this process may not be a chemical mechanism. Regular dental visits, maintenance of good oral hygiene, and reduction in the consumption of betel quid, additives, and cigarettes are highly recommended to improve the periodontal status.

## Background

Betel quid is the fourth most psychoactive substance worldwide [1]. It is usually added to the flower and/or leaf of Piper betle along with several other chemical additives; the mixture is known as betel quid. Betel quid chewing has been associated with oral submucous fibrosis, oral cancer [2, 3] as well as cancer of the esophagus, liver, pancreas, larynx, and lungs; type 2 diabetes mellitus; cardiovascular disease; and adverse pregnancy outcomes [3–7].

The chemical composition of betel quid varies [1, 8], and the effect of factors related to betel quid chewing on the periodontal status are complex [9]. Several *in vitro* studies have suggested that areca nut extracts inhibit immune reactions [10–12], indirectly increase gingival inflammation [13, 14], affect osteoblast viability [15], and may be cytotoxic to periodontal fibroblasts [16]; however, the results of epidemiological studies are not convergent. Hospital-based studies have reported that betel quid chewing is associated with a high prevalence of bleeding on sulcus probing [17] and that betel quid additives may significantly aggravate periodontitis [18]. In contrast, another study reported that betel quid additives have no significant association with chronic periodontitis after adjustment for smoking habits [19]. The results from community-based studies are also inconsistent. Betel quid chewing showed no significant effect on clinical attachment loss after adjusting for oral hygiene in a cross-sectional study [20] and a longitudinal study [21], while it was reportedly a contributing factor to tooth loss in certain cross-sectional studies [22, 23]; in contrast, it was found to be a protecting factor in another longitudinal study [24].

Many factors could have caused the discrespancies in the results of the above investigations. First, tobacco use or cigarette smoking is a confounding factor [25, 26]. Betel quid chewing is almost all coexistent with tobacco usage among men in India [1]. In Taiwan, only 2% male betel quid chewers are nonsmokers [3]. Second, betel quid additives are many and varied. Third, periodontitis is associated with a group of risk factors [9].

To our knowledge, no study has reported the association between betel quid chewing and radiographic alveolar bone loss (RABL). Aboriginal communities in Taiwan comprise a relatively high percentage of nonsmoking betel quid chewers, thereby constituting a suitable population to examine the relationship between betel quid chewing and RABL. The level of consumption and betel quid additives, sociodemographic, lifestyle, and oral hygiene behavioral variables were also investigated.

## Methods

### Ethics and informed consent

The present study was part of a project approved by the Human Experiment and Ethics Committee of Kaohsiung Medical University. Informed written consent was given by all subjects prior to the study. Subjects were informed about any diagnosed pathological findings and were given treatment immediately or were scheduled for further treatment in the public health station.

### Study samples

The subjects were recruited form the aboriginal community, Mutan. Between 2010 and 2011, all subjects belonging to the Paiwan tribe near the Mutan public health station were invited to the station for removal of tooth stains and ultrasonic scaling. Eligibility criteria included no known systemic medical condition such as diabetes mellitus or pregnancy, no dental visits within the preceding 6 months, no consumption of drugs known to affect the periodontium, age between 30 and 60 years, a body mass index of 18.5–25.0, and right-handedness (for evaluation of differences between the right and left sides). Retrospective radiographs (Kodak DF-58, Ultra-speed periapical film; Eastman, Kodak Co., Ltd., Rochester, NY, USA) from 2000 to 2009 were used to detect proximal caries. The paralleling technique was mainly used for the anterior teeth, while the vertical bitewing technique was mainly used for the posterior teeth using XCP® film holders (Rinn, Elgin, IL, USA) Radiographs were automatically processed at room temperature (Perio-Pro automatic processors, Pearson Dental Supply Co.) in a freshly mixed developer and fixer (Kodak RP X-Omat developer and replenisher; Kodak RP X-Omat Lo, fixer and replenisher).

### Questionnaire-based interviews

The subjects were interviewed and data were collected on sociodemographic variables, i.e., age, gender, occupation, education, and ethnicity; lifestyle; i.e., a history of betel quid chewing and smoking (including the number of units consumed per day), the duration of the habit (years), and the additives consumed and oral hygiene practices, i.e., frequency and methods of tooth brushing.

### Clinical examination

Visible bacterial plaque and gingival inflammation were assessed according to the plaque index (PI) [27] and gingival index (GI) [28], respectively, on the facial and palatal/lingual aspects of all existing teeth, except the third molars.

### Radiographic examination

Radiographic examination was performed using the digital scanning radiographic image analysis method (DSRIA) [29], and RABL was defined as the distance between the highest alveolar bone ridge adjacent to the tooth surface and the cementoenamel junction. Reliability was confirmed over a 4-week period by double-reading of 30 molars, 30 canines, and 30 incisors selected randomly, and the reliability coefficients [30] were 0.92, 0.83, and 0.90, respectively. Interexaminer reliability was assessed through replicate readings of 60 sites, and the reliability coefficient was 0.88 for assessing RABL.

### Statistical analysis

Least-square means were used to adjust the measurements by variables and were compared among different subgroups. Descriptive statistics were calculated for relative means, and the differences were tested between groups. The radiographic measurements of tilted, missing, or elongated teeth as well as those of teeth with a large restoration, prosthesis, or apparent root fractures were recorded as missing data. Data were recorded as missing if the chewing and smoking habits were irregular or the question was not answered, particularly in the multiple regression model. Multiple regression analysis was used to assess the relationship between potential risk factors and RABL. Finally, the participants were divided into three groups as follows: betel quid chewers with a smoking habit (smoker and chewer group), betel quid chewers with no smoking habit (chewer group), and subjects with no chewing or smoking habits (control group). All statistical analyses were performed using SAS v9.13 software (SAS Institute Inc., Cary, NC, USA).

## Results

In total, 114 subjects (mean age, 43.3 ± 6.1 years; range, 30–60 years) were included in this study. The chewer group as well as the smoker and chewer group included 41 males and 37 females, while the control group included 11 males and 25 females (Table 1). The adjusted mean RABL in the chewer group as well as the smoker and chewer group showed significant differences with regard to age.

**Table 1:** **Comparison of the adjusted mean RABL of all chewer and control group adjusted by age and gender according to different education level, dental visiting habit and frequency of dental cleaning**

	All chewer		Control group	
	***N*** (%)	Adjusted means (CI)	***N*** (%)	Adjusted means (CI)
Age				
51 to 60 years old	13 (16.7)	4.25 (3.68-4.83)*^†^	3 (8.3)	2.68 (1.88-3.48)^†^
41 to 50 years old	43 (55.1)	3.73 (3.42-4.03)	16 (44.4)	2.77 (2.42-3.12)
30 to 40 years old	22 (28.2)	2.98 (2.55-3.41)	17 (47.2)	2.64 (2.30-2.98)
Gender				
Male	41 (53.6)	3.82 (3.52-4.13)^‡^	11 (30.6)	2.95 (2.54-3.35)^‡^
Female	37 (46.4)	3.34 (3.03-3.67)	25 (69.4)	2.59 (2.32-2.86)

CI, confidence interval.

*Testing equivalence among groups, *p* < 0.05.

^†^Gender-adjusted alone without age adjustment.

^‡^Age-adjusted alone without gender adjustment.

Age- and gender-adjusted mean RABL also showed significant differences between the chewer group as well as the smoker and chewer group and the control group with regard to the chewing pattern, chewing quantity, and betel quid additives (Table 2). Compared with the nonchewers, the adjusted mean RABL was significantly higher if there was a daily chewing habit, if >10 pieces were chewed per day, if the chewing habit persisted for >10 years, if >33,000 pieces were chewed till the present date, or if betel fruit, betel leaves, or lime was used with betel quid. The frequency of chewing, duration, and cumulative amount exhibited dose–response characteristics.

**Table 2:** **The means RABL of different betel quid chewing habit with or without adjusting by age and gender**

	***N***(%)	Means ± SD	Adjusted means (CI)
Control group	36	2.70 ± 0.68	2.87 (2.56-3.18)
Frequency of chewing/week			
7 days/week	47 (75.8)	3.79 ± 1.19*	3.65 (3.38-3.92)*
4-6 days/week	7 (11.3)	3.12 ± 0.70	3.17 (2.49-3.84)
1-3 days/week	8 (12.9)	2.93 ± 0.68	3.13 (2.49-3.77)
Daily chewing habit			
> = 20 pieces/day	16 (32.7)	3.96 ± 1.41*	3.82 (3.37-4.26)*
10-19 pieces/day	20 (40.8)	3.39 ± 0.79	3.29 (2.89-3.68)*
1-9 pieces/day	13 (26.5)	3.37 ± 1.39	3.47 (2.96-3.98)
Duration of intake			
>20 years	30 (44.6)	4.07 ± 1.33*^†^	3.85 (3.51-4.19)*^†^
10-20 years	18 (27.7)	3.50 ± 0.71*	3.56 (3.15-3.98)*
<10 years	18 (27.7)	2.91 ± 0.52	2.98 (2.56-3.39)
Cumulative amount			
>100 k pieces	19 (39.6)	4.07 ± 1.33*^‡^	3.84 (3.44-4.24)*^‡^
33 k-100 k pieces	14 (29.1)	3.71 ± 1.10*^‡^	3.69 (3.23-4.16)*^‡^
0-33 k pieces	15 (31.2)	2.77 ± 0.58	2.92 (2.48-3.35)
Areca nut additives (multiple choices)			
White lime, leaf of betel pepper	43 (55.1)	3.86 ± 1.24*	3.75 (3.46-4.04)*
Red lime, fruit of betel pepper	10 (12.8)	3.86 ± 1.62*	3.67 (3.02-4.32)*
Stem of betel pepper	14 (17.9)	3.34 ± 0.86*	3.35 (2.95-3.76)*
No additives	4 (5.1)	3.16 ± 0.78	3.12 (2.44-3.79)
Other	2 (2.6)	3.77 ± 1.54	3.59 (2.61-4.57)

SD, standard deviation; CI, confidence interval.

*Significant compare with control group.

^†^Significant compare with “<10 years”.

^‡^Significant compare with “0-33 k pieces”.

Oral hygiene was a contributory factor associated with the periodontal status. After adjustment for age, gender, and PI, the full-mouth mean RABL was significantly higher in the smoker and chewer group as well as the chewer group than in the control group; furthermore, it was comparable with the mean RABL obtained on the basis of the Ramfjord tooth index in all three groups (Table 3).

**Table 3:** **The means and adjusted means of RABL adjusted by age, gender and plaque index according to different teeth group**

	Means ± SD	Adjusted means (CI)
Full mouth		
Smoker and chewer	3.73 (1.09)*^†^	3.62 (3.30 - 3.94)*^†^
Chewer	3.46 (1.10)^‡^	3.47 (3.15 - 3.79)^‡^
Control group	2.70 (0.68)	2.87 (2.56 - 3.18)
Ramfjord Teeth		
Smoker and chewer	3.81 (1.16)*^†^	3.68 (3.35 - 4.01)*^†^
Chewer	3.53 (1.17)^‡^	3.56 (3.22 - 3.89)^‡^
Control group	2.75 (0.63)	2.92 (2.60 - 3.25)

SD, standard deviation; CI, confidence interval.

*Testing equivalence among groups, *p* < 0.05.

^†^Significant pair between smoker/chewer and control group.

^‡^Significant pair between chewer and control group.

Only 47 regular chewers and 36 nonchewers were included in regression analysis. In the multiple regression for RABL, age, PI, and cumulative amount of betel quid intake were significant contributing factors (Table 4). The gender variable was excluded because most smokers and chewers were males and most chewers were females. Mean RABL increased by 0.40 mm (*p* = 0.03) for every 100,000 pieces chewed in the chewer group and 0.34 mm (*p* < 0.01) in the smoker and chewer group.

**Figure 1:**
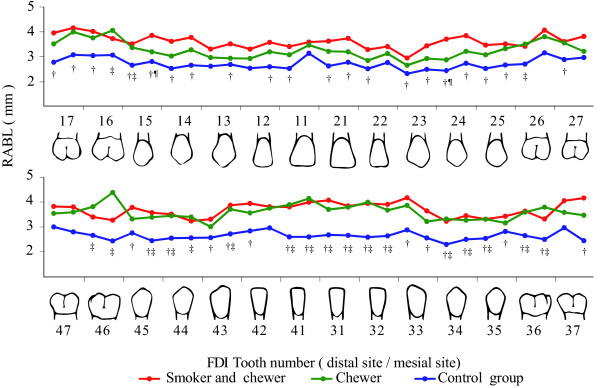
**The means of radiographic alveolar bone loss.** Significant pairs: ^†^smoker and chewer with control group, ^‡^chewer with control group and ^¶^smoker and chewer with control group, *p* < 0.05.

**Figure 2:**
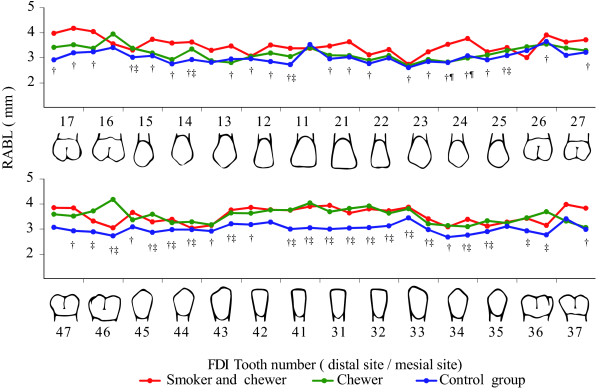
**The adjusted means of radiographic alveolar bone loss adjusted by age, gender and plaque index.** Significant pairs: ^†^smoker and chewer with control group, ^‡^chewer with control group and ^¶^smoker and chewer with control group, *p* < 0.05.

The data for full-mouth mean RABL are shown in Figure 1. In the maxilla, the mean RABL in the chewer group was lower than that in the smoker and chewer group but higher than that in the control group. After adjustment for age, gender, and mean PI, the mean RABL in the chewer group was similar to that in the control group in the maxilla and similar to that in the smoker and chewer group in the mandible, with the exception of RABL around the mandibular right first molar (Figure 2). The mean RABL in all chewers was significantly higher than that in the control group in the mandibular anterior region.

**Table 4:** **Multiple regression for RABL**

	Estimate (mm)	Standard error	T for H _0_: Parameter = 0	***P***-value
Age	0.05	0.01	3.79	<0.01
Means of PI	0.59	0.13	4.39	<.001
Cumulative amount of areca nut intake (100,000 pieces)				
Smoker and chewer	0.34	0.12	2.95	<0.01
Chewer	0.40	0.18	2.22	0.03

*R* ^2^ = 0.51, F = 20.25.

## Discussion

Intraoral radiography provides information on the height and configuration of the interproximal alveolar bone; however, it is not sufficient to identify the outline of the buccal and lingual alveolar bony crest [26]. Although intraoral radiography plays only a supplementary role in the evaluation of the periodontal status in conjunction with periodontal probing, it has high sensitivity, yields few false-negative results, and has high reproducibility compared with the periodontal chart-based RABL evaluation used in previous clinical studies [26, 31]. Although the effects of betel quid chewing on periodontal disease are clinically obvious, research results are controversial. The present study is the first to investigate the association between betel quid chewing and RABL.

The present study showed that after adjustment for age, gender, and PI, the mean RABL in the smoker and chewer group as well as the chewer group was significantly higher than that in the control group, showing an upward trend in RABL with older age, cigarette smoking, poor oral hygiene, higher consumption of betel quid, and consumption of extra additives with betel quid. Furthermore, areca nut chewing without additives may not be the major risk factor of oral submucous fibrosis [32]. Plotted curves for adjusted mean RABL in the maxilla were similar between the chewer and control groups, with the exception of RABL around the mesial root of the right maxillary molar (Figure 2). These findings may indicate that chemical effects were not the main contributory factors affecting the association between betel quid chewing and the periodontal status, suggesting that the influence of chemical or mechanical factors should be identical for both the maxilla and mandible. This finding also implied that specific details of the results may have been submerged after averaging the measurements; therefore, the true result could be observed first after full-mouth analysis. Extensive and heavy calculus deposition is usually observed in the cervical area of the mandibular teeth of chewers [33]. In the present study, this greatly exceeded the existing measurement index and could not be recorded accurately. Excessive calculus deposition can result in an altered microflora [17] and mechanical interference with soft tissues. Further research is required to modify PI or develop a new oral hygiene index that can adequately describe the oral hygiene status of chewers.

After grouping the cumulative amounts in three levels, we found a significant dose–response of betel quid chewing with RABL. To our knowledge, this is the first investigation to demonstrate the cumulative effect of chewing quantity. Chewing more than 33,000 pieces will lead to significant RABL and dose–response confirmed by multiple regression analysis; however, mean PI and age were stronger contributing factors. Multiple regression analysis showed that the chewer group only had 0.40 mm (*p* = 0.03) mean RABL after chewing 100,000 pieces of betel quid. It appears as a clinically insignificant value but also means that the chewer group had 22.4 mm more RABL than the control group in total.

For decades, studies have shown that smoking (both tobacco and cigarette usage) is a major risk factor for periodontitis [25, 34, 35]. For the maxilla, the plotted curves for adjusted mean RABL in the smoker and chewer group were steeper than those in the chewer group and similar to those in the control group, whereas for the mandible, the curves were similar between the chewer group and the chewer and smoker group. It was observed that cigarette smoking primarily affected RABL in the maxilla, whereas betel quid chewing primarily affected RABL in the mandible. Therefore, a combination of chewing and cigarette smoking is associated with a risk of significant clinical periodontal problems.

Women chew betel quid during social events and traditional activities, whereas men chew them for refreshment while working. This behavioral difference may affect the mean duration of chewing the same quantity. We also noted several apparent vertical root fractures with intact crowns in the radiographs of chewers, which were not noted in the control group. In the present study, most participants in the chewer group were female, and the high level of RABL around the right first molars of these subjects may have resulted from the longer duration of chewing force compared with that in men.

This study had several limitations. First, although all residents were invited, individuals who either did not pay attention to oral hygiene or were almost edentulous may not have responded to our invitation. Second, retrospective radiographs cannot provide conclusive evidence of any significant correlation between betel quid chewing and alveolar bone loss; however, dose–response effects support the possibility of a causal relation. Furthermore, several other factors that may be correlated with alveolar bone height were not examined in this study, such as microorganism profiles and blood chemistry factors.

## Conclusions

The present study indicated that betel quid chewing is a contributory factor associated with RABL. RABL was significantly higher in the chewer group as well as the smoker and chewer group than in the control group. Additives and cumulative consumption had dose–response effects on RABL. The main contributory factor associated with alveolar bone loss caused by betel quid chewing may be uncontrolled heavy deposition rather than any chemical or mechanical factors. Regular dental visits, maintenance of good oral hygiene, and reduction in the consumption of betel quid, additives, and cigarettes are highly recommended to improve the periodontal status.
